# Precursor B-cell acute lymphoblastic leukemia presenting as obstructive jaundice: a case report

**DOI:** 10.1186/1752-1947-5-269

**Published:** 2011-07-01

**Authors:** Muhammad N Siddique, Muhammad Popalzai, Nelly Aoun, Rabih Maroun, Michael Awasum, Qun Dai

**Affiliations:** 1 Department of Medicine, Staten Island University Hospital, 475 Seaview Avenue, Staten Island, NY 10305, USA; 2 Department of Hematology/Oncology, Staten Island University Hospital, 475 Seaview Avenue, Staten Island, NY 10305, USA; 3 Department of Pathology, Staten Island University Hospital, 475 Seaview Avenue, Staten Island, NY 10305, USA

## Abstract

**Introduction:**

Acute leukemias very rarely present with jaundice. Herein we report a case of precursor B-cell acute lymphoblastic leukemia that presented with jaundice in an adult.

**Case presentation:**

A 44-year-old Hispanic man presented with right upper quadrant abdominal pain and jaundice. His initial blood work revealed pancytopenia and hyperbilirubinemia. Direct bilirubin was more than 50% of the total. His imaging studies were unremarkable except for hepatomegaly. All blood screening tests for various hepatocellular etiologies were normal. A diagnosis of precursor B-cell acute lymphoblastic leukemia was made upon liver biopsy. It also showed lymphocytic infiltration of the hepatic parenchyma leading to bile stasis. The diagnosis was subsequently confirmed upon bone marrow biopsy. The patient was treated with a hyperfractionated cyclophosphamide/vincristine/doxorubicin/dexamethasone regimen.

**Conclusion:**

Acute lymphoblastic leukemia should be one of the differential diagnoses that should be considered when initial work-up for jaundice is inconclusive. Some cases of acute lymphoblastic leukemia have been reported in both adults and children to have presented with the initial manifestation of jaundice, but only a few had no radiographic evidence of biliary obstruction. Such presentation can pose a serious diagnostic dilemma for clinicians. This manuscript attempts to highlight it. Moreover, we believe that if acute lymphoblastic leukemia presentations similar to this case continue to be reported in adults or children, a specific immunophenotypic expression and cytogenetic abnormality may be found to be associated with hepatic infiltration by leukemia. This may substantially contribute to the further understanding of the pathophysiology of this hematologic disease.

## Introduction

Acute lymphoblastic leukemia (ALL) is a clonal hematologic disorder. It involves excessive proliferation and impaired differentiation of leukemic blasts that lead to inadequate normal hematopoiesis. Thus patients usually present with symptoms resulting from bone marrow failure. The extra-medullary form of this disease is rarely reported. However, when found, it most commonly involves the bones, followed by soft tissue, skin and lymph nodes. It is extremely rare for patients with ALL to present with hepatic manifestations. Herein we present a case of precursor B-cell (pre-B-cell) ALL that manifested as obstructive jaundice. The case elaborates this unique presentation and that infiltrative involvement of leukemia should be considered when the initial work-up for obstructive jaundice is inconclusive. Moreover, it highlights the challenges in planning chemotherapeutic treatment in the presence of an already compromised hepatic function.

## Case presentation

A 44-year-old Hispanic man presented to our hospital with the chief complaints of pain in the right upper quadrant of the abdomen and jaundice. These symptoms were associated with intermittent nausea and vomiting, generalized weakness, poor appetite, clay-colored stools and mild, generalized itching. The patient's symptoms had developed gradually and had worsened over the course of a few weeks. He denied a history of fever or chills or a change in bowel habits. He did report weight loss of about 20 pounds during the six months prior to presentation. It was not entirely unintentional, however, as he was attempting to lose some weight. This symptom was not associated with night sweats. The rest of the review of the patient's systems was unremarkable.

His medical history included diabetes mellitus and hypertension. His social history was significant for smoking. He had quit alcohol intake five months before his presentation to our hospital. He denied any intravenous drug use, recent travel history or exposure to any people who were ill. He did not report any recent change in his medications and was tolerating his aspirin, sitagliptin, fosinopril, metformin and repaglinide without reported side effects. His physical examination was significant for pallor, icteric sclerae and non-tender hepatomegaly. His vital signs were normal. His body temperature was 98°F, his blood pressure was 125/78 mmHg, his pulse was 76 beats/minute and his respiratory rate was 16 breaths/minute. The patient was admitted to the medical service for further work-up.

A complete blood count was significant for pancytopenia, with hemoglobin 8.9 g/dL, white blood cell count 3600/mm^3 ^and platelet count 94,000/mm^3^. His chemistry panel revealed hyperbilirubinemia, with total bilirubin 10 mg/dL, direct bilirubin 7 mg/dL and only minimal elevation of transaminases (alanine transaminase 74I U/L and aspartate transaminase 52I U/L). His alkaline phosphatase and γ-glutamyl transferase levels were significantly raised at 293I U/L and 327I U/L, respectively. This profile is most consistent with obstructive jaundice. However, to rule out hepatocellular causes in this patient, we requested screens for hepatitis A immunoglobulin M (IgM) antibody, hepatitis B surface antigen, anti-nuclear antibodies, anti-smooth muscle antibodies, anti-mitochondrial antibodies, peri-nuclear anti-neutrophil cytoplasmic antibodies and human immunodeficiency virus 1/2 antibodies, and all serologies came back negative. The patient's serum levels of α-fetoprotein and CA 19-9 were also found to be within normal limits. His coagulation profile and electrolytes were normal.

**Figure 1:**
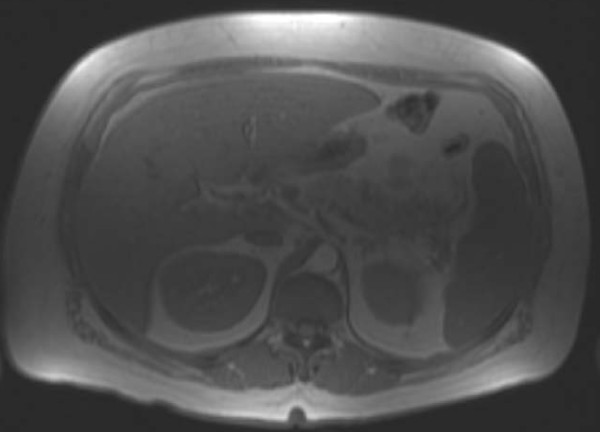
**Magnetic resonance imaging scan of the liver with contrast enhancement obtained during the first week of admission showing a normal hepatobiliary tree**.

About two weeks prior to presentation this patient had undergone magnetic resonance cholangiopancreaticography at another hospital. The scans did not show any intra-or extra-hepatic biliary duct dilatation. Magnetic resonance imaging of the liver with intravenous contrast enhancement done at our hospital revealed hepatomegaly. A right upper quadrant sonogram confirmed the presence of hepatomegaly with increased parenchymal echogenicity, suggestive of fatty infiltration or hepatocellular disease. These imaging studies did not show any evidence of intra-or extra-hepatic biliary duct dilatation and thus failed to fully explain the obstructive jaundice with a bilirubin level of 10 mg/dL (Figure 1).

The patient's pancytopenia was also further explored and was best attributed to the possibility of hypoproliferative marrow, as his reticulocyte production index came back low (0.83), his direct Coombs test was negative and his haptoglobin level was normal (262 mg/dL). However, interestingly, his lactate dehydrogenase level was raised (319I U/L).

As his initial blood work and radiological investigations were inconclusive, an ultrasound-guided core liver biopsy was performed. The histopathology revealed lymphocytes infiltrating the hepatic parenchyma. His immunohistochemistry (IHC) was positive for CD45, CD10, terminal deoxynucleotidyl transferase (TdT), CD79A and PAX5 in the large cell infiltrate. CD20, CD3 and CD5 were negative in the infiltrate but were found to be positive in the rare small lymphocytes. Bcl2 was also faintly positive. CD117, CD23, pan-melanoma marker and pan-cytokeratin were all negative. The myeloid cell markers CD13, CD15 and myeloperoxidase were also negative. This IHC staining pattern was most compatible with a diagnosis of pre-B-cell ALL. Besides this evidence, his hepatic parenchyma showed changes consistent with bile stasis. The patient's liver biopsy was thus suggestive of obstructive jaundice secondary to infiltration of liver parenchyma with leukemic cells.

**Figure 2:**
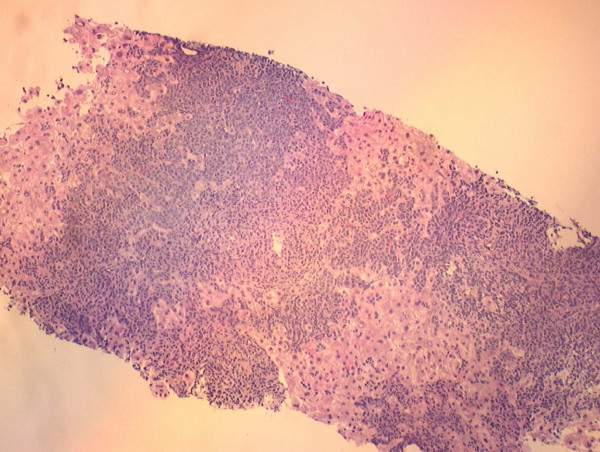
**Ultrasound-guided core biopsy of the liver showing small to medium-sized lymphoblasts infiltrating the hepatic parenchyma**.

**Figure 3:**
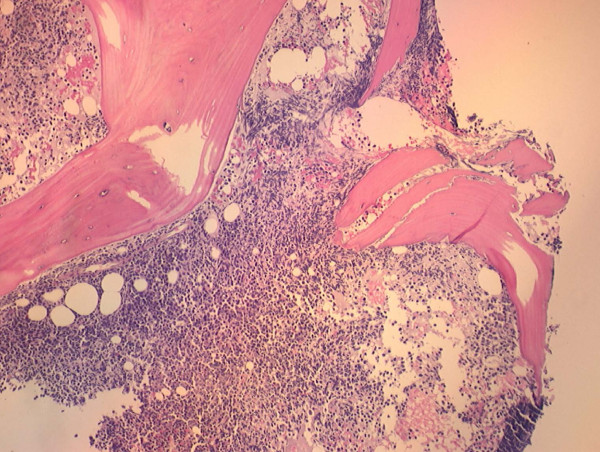
**Bone marrow biopsy showing lymphoblastic infiltration**.

This work-up was followed by a bone marrow biopsy, which confirmed the diagnosis of pre-B-cell ALL. It showed > 90% cellularity with a diffuse, uniform infiltration of lymphoid blasts that had prominent nucleoli. Normal cell lines (erythroid, myeloid and megakaryocytic) were markedly decreased. IHC staining was positive for CD34, CD10 and TdT. Rare small lymphocytes were positive for CD3, CD5 and CD20. His IHC was negative for CD117, CD23, cyclin D1 and myeloperoxidase. Flow cytometric immunophenotypic analysis of his bone marrow aspirate revealed 62% lymphoblasts in the bone marrow, with the following phenotypes: CD34-positive, TdT-positive, CD19-positive, CD79a-positive, CD10-positive (bright), CD45-positive (dim to moderate) and CD20-negative, and cytoplasmic IgM was not expressed. Dim to moderate positivity of CD13 was identified. CD33 was negative. Cytogenetic studies showed an additional copy of chromosomes X and 8 in 7 of 20 mitotic cells. *BCR/ABL *rearrangement was not found. Cerebrospinal fluid cytology and flow cytometry did not reveal any evidence of leptomeningeal involvement (Figures 2 and 3).

We planned to initiate a hyper-CVAD protocol (hyperfractionated cyclophosphamide/vincristine/doxorubicin/dexamethasone) as an induction therapy. As the patient's serum bilirubin level had elevated up to a very high level of 13 mg/dL by that time, it was decided to administer corticosteroids prior to chemotherapy. Prednisone 100 mg every 12 hours was administered for five days before hyper-CVAD therapy was initiated. Day four chemotherapy had to be omitted because of the patient's bilirubin level of 11 mg/dL. By day 11, his bilirubin had dropped to 4 mg/dL, and vincristine was administered with a 50% dose reduction. The day four doxorubicin that had been omitted was also administered with a 50% dose reduction on day 11. As central nervous system prophylaxis, intra-thecal methotrexate was administered on the same day. His bilirubin level continued to fall gradually. It had completely normalized by the time cycle 2 of hyper-CVAD therapy was begun. The patient tolerated the chemotherapy fairly well, except that he required intermittent packed red blood cells and platelet transfusions to support his cell counts and granulocyte colony-stimulating factor and prophylactic intravenous antibiotics for neutropenia. He was discharged after the recovery of his cell counts with instructions to follow up with the hematology/oncology department as an out-patient.

## Discussion

Liver involvement by hematologic malignancies is not infrequent, though it is rarely the initial presentation. Histopathological examinations of the liver specimens in an autopsy series revealed that 44% of untreated patients with leukemias and lymphomas and 26% of the specimens from patients who received chemotherapy either alone or in combination with radiotherapy had evidence of neoplastic involvement [1].

Several patterns of hepatic involvement in hematologic malignancies have been described in the literature. It varies from an asymptomatic hepatic lesion or hepatomegaly to liver failure. Four cases of fulminant hepatic failure were reported by Zafrani *et al*. [2]. These patients had moderate to severe infiltration of the liver parenchyma by leukemic cells with or without accompanying hepatic necrosis. Rarely, obstructive jaundice is the initial presentation. Lee *et al*. [3] described a case of AML with granulocytic sarcoma obstructing the biliary tract. Granulocytic sarcoma represents masses of granulocytic cells. The presence of myeloperoxidase in these myeloid cells gives these masses a greenish coloration and therefore they were historically termed as chloromas [4].

Obstructive jaundice is a very rare presentation of ALLs. Some cases of T-cell ALL [5] and B-cell ALL [6,7] have been reported to have presented in association with jaundice. Only a few of these cases had no evidence of biliary obstruction on imaging. The pathophysiology of jaundice in these cases of ALL was leukemic infiltration of hepatic sinusoids. This is similar to the pathophysiology of jaundice in our case and is in contrast to how AML has been reported to cause jaundice, as reported by Lee et al and as referred to above.

Hepatic insufficiency at presentation of malignancies may pose an important therapeutic challenge as it may reduce tolerance to intensified chemotherapy. Biliary drainage procedures may prove helpful prior to administering chemotherapy in patients with biliary obstruction but may not be possible in patients with diffuse infiltration of hepatic parenchyma by leukemic blast cells [8]. Some authors have suggested a short course of prednisone prior to instituting full dose chemotherapy [9]. Once a downward trend in bilirubin level is established, one can proceed with further chemotherapy [10]. We followed a similar plan for our patient. In our patient, pre-treatment with prednisone resulted in reduction of the bilirubin after which hyper-CVAD was administered. We continue to follow this patient and plan to publish more about his disease in the event it takes an unusual course.

## Conclusion

In this description of an unusual presentation of ALL in our patient, we have attempted to emphasize that hematologic malignancies, especially ALL, should be considered in the differential diagnosis of jaundice. Moreover, we believe it would be interesting to study the disease characteristics of ALL in this specific subset of patients if similar cases continue to be reported. Identification of a pattern of specific cytogenetic abnormalities and immunophenotypic expression associated with such cases may help clinicians to understand the pathogenesis of the disease's progression in general and that of liver involvement in particular. This is why detailed results of IHC and flow cytometry for our patient are being published here.

## Abbreviations

Hyper-CVAD: hyperfractionated cyclophosphamide/vincristine/doxorubicin/dexamethasone; IHC: immunohistochemistry; pre-B-cell ALL: precursor B-cell acute lymphoblastic leukemia.

## Consent

Written informed consent was obtained from the patient for publication of this case report and any accompanying images. A copy of the written consent is available for review by the Editor-in-Chief of this journal.

## Competing interests

The authors declare that they have no competing interests.

## Authors' contributions

MNS was in the patient's care and was a major contributor in writing the abstract, case presentation and conclusion (as the primary author). MP was a major contributor to the Discussion section. NA was involved in the patient's care (as an oncology fellow) and supervised the drafting of the manuscript. RM was involved in the patient's care as the medical attending physician and was a contributor to the Conclusions section. MA performed the histopathology of liver and bone marrow. QD was involved with the patient's care as the attending hematology/oncology physician and made critical revisions of the manuscript. It is hereby certified that all coauthors have seen and agree with the contents of the manuscript and that (aside from abstracts) the manuscript is not under review by any other publication.
